# Dose effects of encapsulated butyric acid and zinc on beef feedlot steer growth performance, dietary net energy utilization, rumen morphometrics, small intestine histology, and carcass characteristics

**DOI:** 10.1093/jas/skae240

**Published:** 2024-08-19

**Authors:** Forest L Francis, Warren C Rusche, Doug LaFleur, Jerilyn E Hergenreder, Zachary K Smith

**Affiliations:** Department of Animal Science, South Dakota State University, Brookings, SD 57007, USA; Department of Animal Science, South Dakota State University, Brookings, SD 57007, USA; Kemin Industries Inc., Des Moines, IA 50317, USA; Kemin Industries Inc., Des Moines, IA 50317, USA; Department of Animal Science, South Dakota State University, Brookings, SD 57007, USA

**Keywords:** butyric acid, beef, feedlot, gut health, zinc

## Abstract

The objective of this study was to determine the effects that increasing doses of encapsulated butyric acid and zinc (BZ) have on feedlot steer growth performance, rumen morphometrics and small intestine histology (data not statistically analyzed), dietary net energy utilization, and carcass characteristics. Steers [*n* = 272; shrunk body weight (**BW**) = 360 kg ± 74 kg] were assigned to dietary treatments [0 (CON), 1, 2, or 3 g BZ/kg diet dry matter] in a randomized complete block design (**RCBD**) with pen (*n* = 32 total; *n* = 8 per treatment) as experimental unit. Pens were blocked by cattle source and location within the feedyard. Cattle were fed until visually assessed to have 1.27 cm rib-fat and were shipped for harvest at a commercial beef abattoir. Carcass and liver health data were recorded. A subset of steers (*n* = 8 total; *n* = 2 per treatment) was harvested at the SDSU Meat Laboratory to collect empty body measurements, rumen samples for morphometric analysis, and duodenal and ileal samples for histological analysis to provide context to feeding trial outcomes. Feedlot growth performance data was calculated on a carcass-adjusted basis: hot carcass weight (**HCW**)/0.625. Data were analyzed as a RCBD with fixed effects of BZ inclusion level and block was considered a random effect; pre-planned contrasts for CON vs. BZ, plus linear, and quadratic responses were tested. No differences (*P* ≥ 0.11) were observed for final BW, dry matter intake, average daily gain (**ADG**), feed conversion efficiency (**G:F**), performance calculated dietary net energy, HCW, ribeye area, rib-fat thickness, marbling score, estimated empty body fat, or distribution of USDA yield grade (**YG**) 1, 3, 4, 5, and USDA quality grade among treatments. A tendency (*P* = 0.10) was observed for CON vs. BZ for calculated YG. Tendencies were detected for USDA YG 2 carcass distribution (linear; *P *= 0.07) and for normal and abscessed liver prevalence (quadratic; *P *= 0.08). Dressed yield tended to be greater (*P* = 0.08) for BZ vs. CON and increased with dose (linear; *P *= 0.05). Receiving period shrunk BW, ADG, and G:F was improved (*P* ≤ 0.02) for BZ-supplemented steers compared to CON. Data from this study suggests that the addition of BZ to feedlot finishing diets to improve receiving period growth performance and decreasing the prevalence of abscessed livers should be further investigated.

## Teaser

Results from this trial indicate that the addition of encapsulated butyric acid and zinc to feedlot steer diets may improve growth performance in the receiving period.

## Introduction

Postnatal development of the rumen and intestinal epithelium is stimulated by short-chain fatty acids (**SCFA**; Warner et al., 1956; Hamada et al., 1976). Of the SCFAs attributed to epithelial development, butyric acid has the greatest effect on proliferation of ruminal and intestinal epithelial cells (Sander et al., 1959; Manzanilla et al., 2006). In dairy calves, supplementing butyrate has beneficial results in growth performance and gastrointestinal health including improved daily gain, decreased fecal scouring days, increased ruminal papillae size, elevated mitotic index, and lower apoptotic index (Mentschel et al., 2001; Górka et al., 2009). Additionally, supplementation of calcium butyrate increased duodenal villi height in confinement-fed bulls (Moreira et al., 2016). Thus, the inclusion of butyrate into cattle diets has potential benefits for improving gastrointestinal health and improving growth performance.

Zinc is a mineral that is essential for a plethora of metabolic and enzymatic processes, and is essential for DNA, RNA, protein synthesis, and cell division in mammals (Prasad, 1983; Cousins et al., 2006). Supplementary zinc improves intestinal permeability and upregulates expression of tight junction proteins in the intestines of mammals (Alam et al., 1994; Zhang et al., 2012). In dairy cows subjected to experimental acidosis via a feed restriction protocol, supplementary zinc hydroxychloride improved ileal villus height and ileum mucosal surface area compared to non-supplemented cows (Horst et al, 2020).

Based on the aforementioned research on butyric acid and zinc in the gastrointestinal tract (**GIT**), dietary addition of encapsulated butyric acid and zinc (**BZ**) that is control released into the GIT of feedlot cattle has the potential to improve rumen and intestinal health and subsequently improve performance metrics. Thus, the objective of this study was to determine the effects that increasing doses of an extended-release BZ have on finishing phase growth performance, efficiency of dietary net energy (**NE**) utilization, rumen morphology, small intestine histology, and carcass characteristics in beef steers. Our hypothesis was that feeding BZ in feedlot finishing steer diets would improve cattle growth performance, carcass characteristics, and efficiency of dietary NE utilization.

## Materials and Methods

All procedures involving the use of animals in this experiment were approved by the South Dakota State University Institutional Animal Care and Use Committee (Approval #2101-004E).

### Dietary treatments

This study used 8 replicate pens per treatment and each pen contained 5 to 10 steers (*n* = 68 steers/treatment). Each pen was assigned to one of 4 dietary treatments in a randomized complete block design (blocked by location of pens within the feedyard). Dietary treatments included:

1. 0 g BZ/kg diet dry matter (**DM**) (CON)
2. 1 g BZ/kg diet DM (1BZ)
3. 2 g BZ/kg diet DM (2BZ)
4. 3 g BZ/kg diet DM (3BZ)

The BZ (VilliTECH; Kemin Industries Inc., Des Moines, IA, USA) was manufactured with proprietary spray freezing technology that microencapsulated butyric acid and zinc in a lipid matrix. The manufacturing process forms granules that have an internal 3-dimensional framework of channels that allow for a controlled and sustained release of butyric acid and zinc throughout the rumen and the remainder of the gastrointestinal tract (Poss et al., 2018). The test product was formulated to contain 30% butyric acid and 10% zinc oxide. No tylosin phosphate was fed over the course of this experiment. All diets contained monensin sodium (Rumensin 90; Elanco US, Inc., Greenfield, IN) at 32.08 mg/kg of diet on a dry matter (DM) basis; all diets were fortified with vitamins and minerals to exceed nutrient requirements for finishing beef steers (NASEM, 2016). The supplements for dietary treatment inclusion were manufactured in 2 batches on February 18, 2021, and July 26, 2021, at the SDSU feed mill located in Brookings, SD. The manufactured supplement was stored in woven polypropylene, duffle top, flat bottom bulk bags and shipped to the SDSU Southeast Research Farm (**SERF**) located southwest of Beresford, SD.

### Cattle feeding and management

Two hundred and seventy-two steers [initial shrunk body weight (**BW**) = 360 kg ± 74 kg] from 2 sources: 1) Central South Dakota and 2) Northwest Iowa were purchased at the Sioux Falls Regional Livestock Cattle Auction (Worthing, SD) and transported 38.5 km to the SERF on March 01, 2021; the 2 sources of cattle remained segregated for the entirety of the study. Upon arrival, steers were offered long-stem grass hay and ad libitum access to water. The following morning, steers were delivered a 50% roughage diet (DM basis) consisting of dry-rolled corn (**DRC**), modified distillers grains with solubles (**MDGS**), corn silage, grass hay, and a liquid supplement fed at 2% of payment BW (DM basis).

On March 05, 2021, steers were processed, applied a unique visual identification ear tag, was vaccinated against viral respiratory diseases (Bovi-Shield GOLD 5, Zoetis Services, LLC, Parsippany, NJ) and clostridial species (ULTRABAC 7/SOMUBAC, Zoetis Services, LLC). In addition, an appropriate dose of pour-on moxidectin (Cydectin, Elanco US, Inc.) was applied, and an individual BW was recorded for allotment purposes. During allocation, steers from both sources remained separate and were randomized and blocked according to the same protocol. Steers were ranked by March 05, 2021, BW, then assigned alternatively to treatments 1 to 4. The data set was then resorted by treatment and BW and a pseudo-random assignment to 1 of 12 replicates was performed. This process produced a similar BW distribution within pens. From March 05 to March 08 steers were delivered the same 50% roughage receiving diet at 2% of their payment weight. In the morning of March 08, 2021, steers were weighed again and allocated into treatment pens; test diets were initiated following morning processing. The initial on-test BW was the average of the 2 BW measures collected on March 05, and March 08, 2021. All live BW measures were pencil shrunk 4% to account for digestive tract fill.

Steers were fed in 2 types of confined feeding systems: 1) Steers (*n* = 232) from 6 replicates were fed in 35 m × 14 m open lot dirt pens (9 to 10 steers/pen) with concrete bunks (6 m linear bunk space) and a concrete apron (3 m); 2) Steers (*n* = 40) from 2 replicates were fed in 12.15 m × 4.5 m partially covered concrete-floor pens (5 steers/pen) with concrete bunks (4.5 m linear bunk space). All pens were equipped with heated continuous flow water troughs that were shared between 2 pens. Individual ingredient samples were collected weekly and analyzed for DM [method no. 935.29 (AOAC, 2012)], N [method no. 968.06 (AOAC, 2016); Rapid Max N Exceed, Elementar Americas, Inc., Ronkonkoma, NY], and ash [method no. 942.05 (AOAC, 2012)]. As the major contributor of fat to test diets, MDGS was analyzed for ether extract (**EE**) for MDGS using an Ankom Fat Extractor (XT10; Ankom Technology, Macedon, NY); tabular EE values were used for the remainder of the ingredients (NASEM, 2016). Additionally, as the lowest contributor of neutral detergent fiber (**NDF**) and acid detergent fiber (**ADF**) to test diets, NDF and ADF were estimated to be 3% and 9%, respectively, for DRC; fiber content analysis for all other ingredients was conducted as described by Goering and VanSoest (1970). Actual diet formulation based upon weekly DM determination and feed batching record with tabular energy values according to Preston (2017) is presented in Tables 1, 2, and 3. Steers were fed once daily at 0800 hours and bunks were managed according to a slick bunk management system allowing ad libitum access to feed, with minimal day-to-day variation in feed deliveries. Feed was manufactured in a commercial mixer wagon (6.1 m^3^; Reel Auggie 3120, Kuhn North America, Inc., Brodhead, WI) with a scale resolution of 0.91 kg.

**Table 1.** Diet formulation for days 1 to 14 of feedlot steers fed increasing doses of encapsulated butyric acid and zinc (BZ)

	Days 1 to 7				Days 8 to 14			
	Dietary BZ inclusion, g/kg diet DM				Dietary BZ inclusion, g/kg diet DM			
Items^1^	0	1	2	3	0	1	2	3
Dry-rolled corn, %	20.63	20.63	20.63	20.63	30.14	30.14	30.14	30.14
MDGS^2^, %	19.81	19.81	19.81	19.81	19.64	19.64	19.64	19.64
Soybean hulls, %	1.80	1.19	0.60	0	1.79	1.19	0.60	0
Corn silage, %	28.07	28.07	28.07	28.07	27.02	27.02	27.02	27.02
Grass hay, %	26.22	26.21	26.21	26.21	17.86	17.86	17.86	17.86
Liquid supplement^3^, %	3.47	3.47	3.47	3.47	3.54	3.55	3.54	3.54
Treatment supplement^4^, %	0	0.61	1.20	1.81	0	0.60	1.19	1.79
Dry matter, %	65.59	65.59	65.59	65.59	64.57	64.57	64.57	64.57
Crude protein, %	13.34	13.33	13.31	13.30	13.15	13.14	13.13	13.12
Neutral detergent fiber, %	38.87	38.87	38.87	38.87	33.79	33.78	33.79	33.79
Acid detergent fiber, %	25.63	25.63	25.63	25.63	21.69	21.69	21.69	21.69
Ash, %	7.48	7.49	7.49	7.49	6.69	6.71	6.70	6.71
Ether extract, %	4.13	4.13	4.13	4.13	4.24	4.24	4.24	4.24
Net energy for maintenance, Mcal/kg	1.77	1.77	1.77	1.77	1.85	1.85	1.85	1.85
Net energy for gain, Mcal/kg	1.11	1.11	1.11	1.11	1.19	1.19	1.19	1.19

^1^All items on a DM basis except DM.

^2^Modified distillers grains plus solubles.

^3^Liquid supplement contained (DM basis): 25.87% crude protein, 22.33% non-protein nitrogen, 0.90 Mcal/kg NE for maintenance, 0.64 Mcal/kg NE for gain, 0.76% ether extract, 16.91% Ca, 0.27% P, 1.23% K, 0.21% Mg, 7.20% NaCl, 3.18% Na, 0.44% S, 4.03 ppm Co, 496.98 ppm Cu, 39.93 ppm I, 50.71 ppm EDDI, 65.64 ppm Fe, 828.31 ppm Mn, 4.03 ppm Se, 2,484.92 ppm Zn, 150,056.65 IU/kg vitamin A, 20,007.78 IU/kg vitamin D3, 825.72 IU/kg vitamin E, and 801.38 mg/kg monensin sodium.

^4^Treatment supplement contained: 86.65% soybean hulls and 13.35% encapsulated butyric acid and zinc.

**Table 2.** Diet formulation for days 15 to 28 of feedlot steers fed increasing doses of encapsulated butyric acid and zinc (BZ)

	Days 15 to 20				Days 21 to 25				Days 26 to 28			
	Dietary BZ inclusion, g/kg diet DM				Dietary BZ inclusion, g/kg diet DM				Dietary BZ inclusion, g/kg diet DM			
Items^1^	0	1	2	3	0	1	2	3	0	1	2	3
Dry-rolled corn, %	38.81	38.81	38.81	38.81	40.07	40.07	40.07	40.07	38.79	38.79	38.78	38.78
MDGS^2^, %	19.75	19.75	19.75	19.75	21.04	21.04	21.04	21.04	22.25	22.25	22.25	22.24
Soybean hulls, %	1.84	1.22	0.61	0	2.45	1.63	0.81	0	2.55	1.70	0.85	0
Corn silage, %	27.08	27.08	27.08	27.08	35.64	35.63	35.63	35.63	32.45	32.45	32.45	32.45
Grass hay, %	8.96	8.96	8.96	8.96	—	—	—	—	—	—	—	—
Liquid supplement^3^, %	3.55	3.55	3.55	3.55	—	—	—	—	3.96	3.96	3.96	3.96
Treatment supplement^4^, %	0	0.62	1.23	1.85	0	0.82	1.64	2.46	0	0.85	1.72	2.57
CTC premix^5^, %	—	—	—	—	0.80	0.80	0.80	0.80	—	—	—	—
Dry matter, %	64.45	64.45	64.45	64.45	61.25	61.25	61.25	61.26	58.04	58.05	58.05	58.06
Crude protein, %	13.00	12.99	12.97	12.96	13.03	13.01	12.99	12.97	14.06	14.04	14.02	14.00
Neutral detergent fiber, %	28.89	28.89	28.90	28.90	27.65	27.66	27.66	27.66	26.75	26.75	26.75	26.76
Acid detergent fiber, %	17.86	17.86	17.86	17.86	14.73	14.73	14.74	14.74	14.35	14.35	14.35	14.35
Ash, %	5.87	5.88	5.88	5.89	3.42	3.43	3.43	3.44	5.68	5.69	5.69	5.70
Ether extract, %	4.35	4.35	4.35	4.35	4.54	4.54	4.54	4.54	4.49	4.49	4.49	4.49
Net energy for maintenance, Mcal/kg	1.93	1.93	1.93	1.93	2.02	2.02	2.02	2.02	1.98	1.98	1.98	1.98
Net energy for gain, Mcal/kg	1.27	1.27	1.27	1.27	1.35	1.35	1.35	1.35	1.33	1.33	1.33	1.33

^1^All items on a DM basis except DM.

^2^Modified distillers grains plus solubles.

^3^Liquid supplement contained (DM basis): 25.87% crude protein, 22.33% non-protein nitrogen, 0.90 Mcal/kg NE for maintenance, 0.64 Mcal/kg NE for gain, 0.76% ether extract, 16.91% Ca, 0.27% P, 1.23% K, 0.21% Mg, 7.20% NaCl, 3.18% Na, 0.44% S, 4.03 ppm Co, 496.98 ppm Cu, 39.93 ppm I, 50.71 ppm EDDI, 65.64 ppm Fe, 828.31 ppm Mn, 4.03 ppm Se, 2,484.92 ppm Zn, 150,056.65 IU/kg vitamin A, 20,007.78 IU/kg vitamin D3, 825.72 IU/kg vitamin E, and 801.38 mg/kg monensin sodium.

^4^Treatment supplement contained: 86.65% soybean hulls and 13.35% encapsulated butyric acid and zinc.

^5^CTC premix contained: 110.23 g/kg chlortetracycline hydrochloride.

**Table 3.** Diet formulation for days 29 to 161 of feedlot steers fed increasing doses of encapsulated butyric acid and zinc (BZ)

	Days 29 to 49				Days 50 to 59				Days 60 to 161			
	Dietary BZ inclusion, g/kg diet DM				Dietary BZ inclusion, g/kg diet DM				Dietary BZ inclusion, g/kg diet DM			
Items^1^	0	1	2	3	0	1	2	3	0	1	2	3
Dry-rolled corn, %	50.31	50.35	50.35	50.31	46.76	46.79	46.79	46.75	60.54	60.58	60.57	60.53
MDGS^2^, %	17.29	17.29	17.29	17.29	17.63	17.63	17.63	17.63	21.09	21.09	21.09	21.09
Soybean hulls, %	2.43	1.63	0.76	0	2.38	1.60	0.74	0	2.52	1.69	0.79	0
Corn silage, %	26.18	26.18	26.18	26.18	26.68	26.68	26.68	26.67	—	—	—	—
Grass hay, %	—	—	—	—	2.83	2.83	2.83	2.83	11.95	11.95	11.95	11.95
Liquid supplement^3^, %	3.79	3.79	3.79	3.79	3.72	3.72	3.71	3.71	3.90	3.90	3.90	3.90
Treatment supplement^4^, %	0	0.76	1.64	2.44	0	0.75	1.62	2.40	0	0.79	1.71	2.54
Dry matter, %	60.53	60.53	60.54	60.54	61.65	61.65	61.65	61.66	77.08	77.09	77.09	77.10
Crude protein, %	13.00	12.98	12.97	12.96	13.09	13.08	13.07	13.06	14.06	14.05	14.04	14.04
Neutral detergent fiber, %	21.62	21.60	21.61	21.63	23.35	23.34	23.34	23.36	21.92	21.90	21.90	21.93
Acid detergent fiber, %	11.57	11.56	11.56	11.58	12.73	12.72	12.72	12.74	12.66	12.64	12.64	12.66
Ash, %	4.89	4.90	4.90	4.91	5.25	5.25	5.26	5.27	5.91	5.91	5.95	5.96
Ether extract, %	4.35	4.35	4.35	4.35	4.33	4.33	4.33	4.33	4.69	4.69	4.69	4.69
Net energy for maintenance, Mcal/kg	2.02	2.02	2.02	2.02	1.99	1.99	1.99	1.99	2.05	2.05	2.05	2.05
Net energy for gain, Mcal/kg	1.36	1.36	1.36	1.36	1.33	1.33	1.33	1.33	1.36	1.36	1.36	1.36

^1^All items on a DM basis except DM.

^2^Modified distillers grains plus solubles.

^3^Liquid supplement contained (DM basis): 25.87% crude protein, 22.33% non-protein nitrogen, 0.90 Mcal/kg NE for maintenance, 0.64 Mcal/kg NE for gain, 0.76% ether extract, 16.91% Ca, 0.27% P, 1.23% K, 0.21% Mg, 7.20% NaCl, 3.18% Na, 0.44% S, 4.03 ppm Co, 496.98 ppm Cu, 39.93 ppm I, 50.71 ppm EDDI, 65.64 ppm Fe, 828.31 ppm Mn, 4.03 ppm Se, 2,484.92 ppm Zn, 150,056.65 IU/kg vitamin A, 20,007.78 IU/kg vitamin D3, 825.72 IU/kg vitamin E, and 801.38 mg/kg monensin sodium.

^4^Treatment supplement contained: 86.65% soybean hulls and 13.35% encapsulated butyric acid and zinc.

On study day 28, steers were weighed, implanted with 200 mg trenbolone acetate and 28 mg estradiol benzoate (SYNOVEX Plus, Zoetis Services LLC) and intranasally vaccinated against Infectious Bovine Rhinotracheitis and Parainfluenza 3 (BOVILIS Nasalgen IP, Intervet Inc., Rahway, NJ). On study day 98, steers were weighed, and unique radio-frequency identification (RFID) ear tags were administered to each animal. Steers from sources 1 and 2 were projected to finish at differing days on feed (**DOF**); thus, ractopamine hydrochloride was fed at a rate of 300 mg/steer/d for the last 28 DOF (source 1; study days 98 to 126) and last 35 DOF (source 2; study days 126 to 161).

### Health management

All steers removed from their home pen for health evaluation were then monitored in individual hospital pens prior to being returned to their home pens. When a steer was moved to a hospital pen, the steers daily allotment of feed (pen intake ÷ pen steer count) from the home pen was removed and transferred to the hospital pen. If the steer in the hospital returned to their home pen, hospital pen feed was credited to the home pen. If the steer did not return to their home pen, all feed that was delivered to the hospital pen was deducted from the feed intake record for its home pen back to the date the steer was hospitalized. Health outcomes were characterized as musculoskeletal (lameness), gastrointestinal (bloat), respiratory (pneumonia), other (pinkeye, etc.), removals (includes animals found dead), and general (dead). One steer from CON was removed from the study because of a broken jaw, and one steer from 2BZ died from heat stress and bloat which was determined to be unrelated to the dietary treatment based on postmortem necropsy by consulting veterinarian. On day 19 of the study, around 3% of the cattle in the yard exhibited signs of respiratory illness. Following examination by the herd veterinarian, metaphylaxis with chlortetracycline was chosen as the best treatment option. Chlortetracycline (Pennchlor 50G, Pharmgate Inc., Wilmington, NC) was fed from days 21 to 25 at a rate of 22.05 mg/kg of BW daily. During this period, liquid supplement was removed from the diet to stay in compliance with medicated feed regulations [i.e., no combination approval with Rumensin 90 (Elanco US, Inc.)].

### Carcass characteristics

Both sources of steers were fed until visually assessed to have 1.27 cm rib-fat and were shipped for harvest at a commercial beef abattoir. Steers from source 1 were weighed off study after 126 DOF, transported to commercial beef abattoir, and harvested the following morning (July 13, 2021). Source 2 steers were weighed off study after 161 DOF, transported to commercial abattoir, and harvested the following morning (August 17, 2021). In the afternoon following final BW determination for the respective sources, steers were transported 98 km to a commercial beef processor for harvest the subsequent morning. Steers were commingled at the time of shipping and remained this way until harvest. For source 1 steers, trained individuals entered the slaughter facility, and individual visual identification tags were recorded, RFID tags were recorded via Allflex RS420NFC Series Stick Reader (Allflex USA LLC, Rahway, NJ), and packer identification tags were recorded to ensure individual carcasses could be traced to live steers. Additionally, livers were visually evaluated to determine health according to the Elanco Liver Check Service (Elanco US, Inc.). For source 2 steers, only one trained individual was allowed to enter the slaughter facility due to SARS-CoV-2 protocols; thus, RFID tags and packer identification tags were recorded to trace carcasses to live animal, and livers were visually evaluated to determine abscess prevalence only (abscessed or healthy). Hot carcass weight (**HCW**) was obtained via plant printouts. Following chilling, all carcasses were ribbed for USDA-AMS grading; quality and yield grade (**YG**) attributes were obtained with camera grading, and kidney, pelvic, and heart fat (KPH) percentage was determined via plant-specific algorithm. Dressing percentage was calculated as: HCW/(final BW shrunk 4%). YG was calculated according to the USDA regression equation (USDA, 2017). Estimated empty body fat (**EBF**) percentage and final BW at 28% EBF (AFBW) were calculated from observed carcass traits (Guiroy et al., 2002).

### Empty body measurements, rumen, and small intestine sample collection

Eight steers were selected from source 1 for harvest at the SDSU Meat Laboratory to collect empty body measurements, rumen samples for morphometric analysis, and ileal and duodenal samples for histological analysis. These measures were quantified to provide context to the observed growth performance responses, but these analyses were not subjected to statistical analysis. One steer from each treatment was selected from replicates 3 and 7 to represent cattle that were fed in open dirt lots and partially covered concrete pens. Steers were transported on the afternoon of July 12, 2021, from the SDSU SERF Feedlot in Beresford, SD to the RNC Feedlot in Brookings, SD for lairage until harvest the following morning. In the morning of July 13, 2021, steers were weighed at the SDSU RNC Feedlot and were transported to the SDSU Meat Lab for harvest. During harvest, viscera were removed and weighed full to obtain a weight including digesta. Digesta was washed from the gastrointestinal tract, and viscera was weighed again to obtain an empty weight. Weight of digesta was subtracted from live weight to obtain empty body weight (**EBW**).

Following evisceration, a rumen sample from the cranial ventral sac was collected and washed with a 0.9% sodium chloride solution to remove any feed particles and was stored in 120 mL urine collection cups in a 70% ethanol solution for further analysis. Small intestine samples were collected from the descending duodenum and the distal ileum, washed with a 0.9% sodium chloride solution, trimmed into ≈4 cm × 2.5 cm sections, placed into histological embedding cassettes, and fixed in 10% neutral buffered formalin.

### Growth performance calculations

Growth performance (live and carcass-adjusted) was calculated on a deads and removals excluded basis. All steers were weighed individually at processing, as well as on days 1, 28, 56, 98, and 126; source 2 cattle were also weighed on day 161. Steers were weighed in a hydraulic squeeze chute mounted on top of load cells (scale readability ± 0.91 kg). Receiving period growth performance data was based upon initial BW (average of March 05 and March 08, 2021, BW) shrunk 4% (SBW) and day 28 SBW. Cumulative growth performance is based upon initial SBW and carcass-adjusted final BW (CAFBW) calculated from HCW/0.625. Average daily gain (**ADG**) was calculated as the difference between day 28 SBW or CAFBW and initial SBW divided by DOF for the respective period; feed conversion efficiency (G:F) was calculated from ADG/dry matter intake (**DMI**).

### Efficiency of dietary NE utilization calculations

Observed dietary NE and the ratio of observed-to-expected dietary NE were assessed for the cumulative feeding period. Carcass-adjusted growth performance was used to calculate performance-based dietary NE to determine efficiency of dietary NE utilization. The performance-based dietary NE was calculated from daily energy gain (**EG**; Mcal/d): EG = (ADG)^1.097^ × 0.0557W^0.75^; where W is the mean equivalent shrunk BW [kg; (NASEM, 1996)] from median feeding SBW (**MBW**) and AFBW calculated as: [MBW × (478/AFBW), kg; (NASEM, 1996)]. Maintenance energy (EM) was calculated by the equation: EM = 0.077 × MBW^0.75^. DMI is related to energy requirements and dietary NE for maintenance (**NEm**; Mcal/kg) according to the following equation: DMI = EG/(0.877NEm − 0.41), and can be resolved for estimation of dietary NEm by means of the quadratic formula x=(-b±b2-4ac)/2a, where *a* = 0.41EM, *b* = –0.877EM + 0.41DMI + EG, and *c* = –0.877DMI (Zinn and Shen, 1998). Dietary NE for gain (NEg; Mcal/kg) was derived from NEm using the following equation: NEg = 0.877NEm − 0.41 (Zinn, 1987). Observed-to-expected (**O:E**) NEm and NEg were a ratio of performance-based dietary NE to tabular dietary NE values (Preston, 2017). The maintenance coefficient (**MQ**) was determined using the following equation: MQ, Mcal/W^0.75^ = [(DMI − (EG/NEg)) × NEm]/MBW^0.75^.

### Rumen morphometric and small intestine histological measurements

From the stored rumen samples, a 1-cm^2^ fragment of each sample was sectioned and papillae were counted to determine papillae per square centimeter (**NOP**). Additionally, 12 papillae were removed from each fragment and scanned (Epson Perfection V30; Seiko Epson Corporation, Tokyo, Japan). Mean papillae area (**MPA**) was determined using an image analysis system (ImageJ; National Institutes of Health, Bethesda, MD), and the area recorded was doubled to account for both sides of the papillae. Rumen wall absorptive surface area (**ASA**) expressed in cm^2^ was calculated as follows: 1 + (NOP × MPA)—(NOP × 0.002), where 1 represents the 1 cm^2^ fragment of rumen and 0.002 represents the estimated basal area of papillae in cm^2^ (Pereira et al. 2021).

Duodenum and ileum samples were transported to the SDSU Animal Disease Research and Diagnostic Laboratory for histological staining. As described above, the samples had been fixed in 10% neutral buffered formalin. The samples were cross-sectioned embedded in paraffin and stained with hematoxylin and eosin for microscopic evaluation. Histological slides were imaged under various magnifications, and digital images were analyzed for villus height, crypt depth, villi-to-crypt ratio, and mucosal thickness with an image analysis system (ImageJ; National Institutes of Health).

### Statistical analysis

Receiving period and cumulative growth performance, carcass characteristics, and efficiency of dietary NE utilization and frequency data were analyzed as a randomized complete block design using the GLIMMIX procedure of SAS 9.4 (SAS Inst. Inc., Cary, NC) with pen as the experimental unit. A fixed effect of BZ inclusion and random effect of block was utilized in model analysis. Interim period performance data was analyzed on a live-basis while cumulative feedlot performance data was analyzed on a carcass-adjusted basis. Pre-planned contrasts for CON vs. BZ, plus linear, and quadratic responses were tested. Least square means were generated with the LSMEANS option of SAS and means were separated and denoted different (*P* ≤ 0.05) using the pairwise comparison PDIFF option of SAS (SAS Inst. Inc.). Significance was determined at (*P* ≤ 0.05) and tendencies were observed at (0.05 < *P* ≤ 0.10). Empty body measurements, rumen morphometrics, and small intestine histological measurements were quantified to provide context to the feeding trial, however, due to a total of only 2 experimental units per treatment these measurements were subjected to statistical analyses only to provide context to the growth performance data and for potential use in future meta-analysis approaches since collecting these types of data are cumbersome and expensive.

## Results

### Growth performance and dietary energetics

Results for receiving period growth performance data are presented in Table 4. No differences (*P* ≥ 0.66) were observed for DMI during the receiving period. A tendency (*P* = 0.09) for a quadratic effect of d 28 shrunk BW was observed. Additionally, there was a tendency (*P* = 0.07) for a linear increase in receiving period ADG with increasing inclusion of BZ in the diet. Day 28 shrunk BW, ADG, and G:F differed (*P* ≤ 0.02) for CON vs. BZ with BZ-supplemented steers producing 1%, 8%, and 7% greater d 28 shrunk BW, ADG, and G:F, respectively, compared to CON cattle. Furthermore, linear increases (*P* ≤ 0.05) for increasing levels of BZ were noted for d 28 shrunk BW and G:F.

**Table 4.** Growth performance of beef feedlot steers supplemented with increasing doses of encapsulated butyric acid and zinc (BZ)

	Dietary BZ inclusion, g/kg diet DM					*P* values		
Items	0	1	2	3	SEM	0 vs. BZ	Linear	Quadratic
Pens, *n*	8	8	8	8	—	—	—	—
Steers, *n*	68	68	68	68	—	—	—	—
Days on feed	144	144	144	144	—	—	—	—
Initial shrunk body weight^1^, kg	360	361	361	361	4.12	0.49	0.92	0.44
Receiving period performance								
Day 28 shrunk body weight^1^, kg	401	406	405	405	4.04	<0.01	0.05	0.09
Average daily gain, kg	1.46	1.58	1.57	1.59	0.061	0.02	0.07	0.22
Dry matter intake, kg	9.29	9.46	9.24	9.38	0.240	0.66	0.94	0.91
Gain:feed	0.157	0.167	0.170	0.169	0.0053	0.02	0.04	0.20
Cumulative carcass-adjusted performance								
Final body weight^2^, kg	635	641	642	641	15.75	0.14	0.22	0.37
Average daily gain, kg	1.92	1.95	1.97	1.96	0.030	0.19	0.24	0.45
Dry matter intake, kg	11.50	11.60	11.57	11.64	0.173	0.37	0.39	0.85
Gain:feed	0.167	0.168	0.171	0.169	0.0030	0.17	0.52	0.52
Observed Dietary Net Energy, Mcal/kg								
Maintenance	2.01	2.02	2.05	2.03	0.020	0.40	0.53	0.41
Gain	1.35	1.37	1.38	1.37	0.020	0.40	0.53	0.41
Observed:expected dietary net energy								
Maintenance	1.00	1.01	1.02	1.01	0.010	0.41	0.53	0.41
Gain	1.01	1.02	1.04	1.02	0.020	0.41	0.53	0.41
Maintenance coefficient (MQ)	0.075	0.073	0.071	0.073	0.0030	0.38	0.50	0.41

^1^Shrunk BW calculated as live BW × 0.96.

^2^Calculated as hot carcass weight ÷ 0.625.

Cumulative feeding period carcass-adjusted growth performance and efficiency of dietary NE results are reported in Table 4. There were no differences (*P* ≥ 0.14) observed for CAFBW, DMI, ADG, G:F, observed diet NEm or NEg, O:E NEm or NEg, or MQ. Least squares means and standard error of the mean for EBW, rumen morphometrics, and small intestine histology are reported in Table 5, however, due to limited replication (*n* = 2 per treatment) probability values are not shown.

**Table 5.** Empty body measures, rumen morphological measures, and small intestine histology of beef feedlot steers supplemented with increasing doses of encapsulated butyric acid and zinc (BZ)

	Dietary BZ inclusion, g/kg diet DM				
Items	0	1	2	3	Variation (SEM)
Steers, *n*	2	2	2	2	—
Live body weight, kg	598	581	596	589	12.5
Empty body measures					
Empty body weight, kg	550	565	561	561	1.5
Empty body weight, % of body weight	93.0	95.7	94.8	94.9	0.28
Rumen morphology^1^					
Papillae count, n/cm^2^ rumen wall	38.8	30.3	33.0	42.2	7.13
Papillae area, cm^2^	0.89	0.97	1.32	0.98	0.133
Absorptive surface area, cm^2^	35.36	30.54	42.78	42.65	8.163
Papillae area, % of ASA	97.35	96.78	97.73	97.66	0.57
Small intestine histology					
Duodenum					
Villi height, μm	530.6	620.3	555.9	575.3	51.30
Crypt depth, μm	225.9	365.9	223.0	277.1	27.96
Villi height:crypt depth	2.35	1.71	2.49	2.08	0.220
Ileum					
Villi height, μm	710.4	623.6	600.0	610.9	53.72
Crypt depth, μm	338.2	269.8	280.4	253.9	25.05
Villi height:crypt depth	2.10	2.32	2.20	2.40	0.258

^1^Rumen morphology measures collected and calculated according to Pereira et al. (2021).

### Carcass characteristics

Carcass characteristics are presented in Table 6. There was no difference (*P* ≥ 0.21) among treatments for HCW, dressed yield, ribeye area, 12th rib-fat thickness, calculated USDA YG, marbling score, or EBF percentage. For CON vs. BZ, there was no difference (*P* ≥ 0.14) for HCW, ribeye area, 12th rib backfat thickness, marbling score, or EBF percentage; CON vs. BZ tended to differ for dressed yield (*P* = 0.08; CON = 64.23% and BZ = 64.86%) and for calculated USDA YG (*P* = 0.10; CON = 2.94 and BZ = 3.06). No quadratic effects (*P* ≥ 0.34) were observed for any variables tested. No linear effect was observed (*P* ≥ 0.15) for HCW, ribeye area, 12th rib backfat thickness, calculated USDA YG, marbling score, or EBF percentage; however, a linear increase was observed (*P* = 0.05) for dressed yield.

**Table 6.** Carcass traits of beef feedlot steers supplemented with increasing doses of encapsulated butyric acid and zinc (BZ)

	Dietary BZ inclusion, g/kg diet DM					*P* values		
Items	0	1	2	3	SEM	0 vs. BZ	Linear	Quadratic
Pens, *n*	8	8	8	8	—	—	—	—
Steers, *n*	68	68	68	68	—	—	—	—
Carcass traits								
Hot carcass weight, kg	397	400	401	401	9.85	0.14	0.22	0.38
Dressed yield^1^, %	64.23	64.55	65.05	64.98	0.30	0.08	0.05	0.53
Ribeye area, cm^2^	92.54	92.25	90.45	91.31	2.08	0.29	0.22	0.56
Rib fat, cm	1.28	1.35	1.33	1.33	0.08	0.29	0.57	0.39
Calculated yield grade	2.94	3.05	3.08	3.07	0.09	0.10	0.15	0.36
Marbling score^2^	422	415	414	417	9.93	0.55	0.68	0.65
Estimated EBF^3^, %	29.39	29.78	29.83	29.73	0.42	0.24	0.40	0.40

^1^Calculated as (hot carcass weight ÷ final BW, shrunk 4%) × 100.

^2^Marbling scores: 400 = Small00 (minimum for USDA choice), 500 = Modest00 (minimum for USDA premium choice).

^3^Empty body fat (EBF) = 17.76207 + (4.68142 × FT) + (0.01945 × HCW) + (0.81855 × QG) − (0.06754 × LMA); FT = fat thickness, cm; HCW = hot carcass weight, km; QG = quality grade (Select = 4, Low Choice = 5, Average Choice = 6, High Choice = 7, Low Prime = 8); LMA = longissimus muscle area, cm^2^ (Guiroy et al., 2002).

### Categorical carcass characteristics

Categorical carcass characteristics can be found in Table 7. There were no treatment differences (*P* ≥ 0.21) for distribution of USDA YG 1, 2, 3, 4, or 5. A tendency for a linear effect (*P* = 0.07) was observed for distribution of USDA YG 2; however, no other linear differences were observed (*P* ≥ 0.25) for distribution of USDA YG 1, 3, 4, or 5. No treatment, CON vs. BZ, linear, or quadratic differences (*P* ≥ 0.68) were observed for distribution of USDA standard, select, low choice, average choice, or high choice. A tendency for a quadratic effect (*P* = 0.08) for liver abscess prevalence was observed; however, no treatment, CON vs. BZ, or linear effects (*P* ≥ 0.32) were observed in the study.

**Table 7.** Categorical carcass outcomes of beef feedlot steers supplemented with increasing doses of encapsulated butyric acid and zinc (BZ)

	Dietary BZ inclusion, g/kg diet DM				*P* values		
Items	0	1	2	3	0 vs. BZ	Linear	Quadratic
Pens, *n*	8	8	8	8	—	—	—
Steers, *n*	68	68	68	68	—	—	—
Distribution of USDA yield grade							
Yield grade 1, %	7.5	4.4	3.0	8.8	0.45	0.95	0.14
Yield grade 2, %	46.3	50.0	41.8	32.4	0.47	0.07	0.27
Yield grade 3, %	41.8	36.8	47.8	48.5	0.73	0.26	0.63
Yield grade 4, %	4.4	4.4	7.4	8.8	0.53	0.25	0.85
Yield grade 5, %	0	4.4	0	1.5	0.99	0.99	0.99
Distribution of USDA quality grade							
Standard, %	0	0	1.5	0	0.99	1.00	0.99
Select, %	44.7	48.5	47.0	45.6	0.78	0.97	0.68
Low choice, %	46.3	42.7	43.9	44.1	0.70	0.85	0.76
Average choice, %	6.0	8.8	6.1	7.4	0.71	0.91	0.83
High choice, %	3.0	0	1.5	2.9	0.97	0.97	0.97
Liver abscess prevalence							
Normal, %	82.1	88.2	88.1	77.9	0.54	0.57	0.08
Abscess, %	17.9	11.8	11.9	22.1	0.54	0.57	0.08

## Discussion

Over the cumulative feeding period, the inclusion of BZ into feedlot steer diets had few effects on growth performance. However, during the receiving period, positive results were realized from the inclusion of BZ into feedlot diets. In feedlot cattle being adapted to a finishing diet, the most common protocol to use is a multiple diet step up system where cattle will gradually receive less forage and more concentrate to allow proper time for rumen bacterial populations to adjust and for rumen papillae to grow (Brown et al., 2006; Millen et al., 2009; Samuelson et al., 2016). Butyric acid is the SCFA most associated with rumen papillary and intestinal villi growth (Sander et al., 1959; Salminen et al., 1998). While no differences were observed for rumen morphometrics at the end of the feeding period, no samples were taken from steers immediately following the receiving period. Thus, it cannot be ruled out that improved growth performance of BZ steers during the receiving period could be from increased rumen papillae and small intestine villus size and an inherent increase in absorptive surface area that was better capable of clearing increases in total SCFA produced during adaptation to the finishing diet. Future research should investigate rumen morphometrics of BZ-supplemented steers following the receiving period. There was limited replication and that more research is needed to confirm these responses. If an increase in absorptive surface area occurred, the enhanced clearance of SCFA’s may have mitigated accumulation of acids and helped to prevent ruminal or hindgut acidotic conditions. These results may not have been realized over the cumulative feeding period because of increases in dietary concentrates, intake, and subsequent increases in total SCFA production in the rumen and lower tract. During the receiving period, the contribution of the supplementary butyric acid to total SCFA pool would have been greater and was likely more useful to the growth of the gastrointestinal tract epithelium during dietary transition than after adaptation to the finishing diet. The increased BW for BZ-supplemented steers during the receiving period was numerically carried through the cumulative finishing period but did not differ (*P* = 0.14) at study termination.

Dressing percentage tended to be increased with the supplementation of BZ in the diet and was linearly increased with increasing BZ inclusion. Historically, dressing percentage and carcass fatness have been highly correlated (Owens et al., 1995). However, in the current study, while calculated YG tended to be higher for BZ-supplemented steers, as evidenced by a tendency for a linear decrease in YG 2 carcasses as BZ inclusion increased, 12th rib-fat thickness was not appreciably different indicating that other carcass cutability measurements are driving the increase. Lofgreen et al. (1962) reported that gastrointestinal fill was a more efficient predictor of dressing percentage than carcass fatness. The BZ-supplemented steers had greater EBW as a percent of live BW than CON, which was evidenced by a 29% decrease in gut fill (data not shown). Less dietary fill as a percentage of BW would yield improvement in dressing percentage as was noted in this study.

A tendency for a quadratic effect on liver abscess prevalence was noted, where steers supplemented 1 and 2 g BZ/kg diet DM had a reduced percentage of liver abscesses compared to control and 3 g BZ/kg diet DM steers. The accepted etiological dogma for liver abscess formation is that acidotic conditions in the rumen cause damage to the rumen epithelium and bacteria, specifically *Fusobacterium necrophorum*, which can pass the epithelial barrier and migrate to the liver and cause a bacterial infection (Amachawadi and Nagaraja, 2022). In the current study, while not statistically significant, papillae area and absorptive surface area of the rumen were 22% and 9% greater, respectively, for BZ-supplemented steers compared to control. With increased absorptive surface area, the clearance of SCFA’s and organic acids in the rumen of supplemented steers may have been greater and better able to reduce acidotic conditions that could lead to liver abscesses. The hindgut has also been implicated in liver abscess formation because of its part in overall gut acidosis syndrome (Amachawadi and Nagaraja, 2022). Compared to the stratified squamous epithelial layer of the reticulorumen, the hindgut’s single columnar epithelium is inherently more susceptible to acidotic conditions and bacterial translocation (Amachawadi and Nagaraja, 2022). Additionally, the hindgut’s buffering capacity is decreased compared to the rumen because of the lack of salivary flow (Amachawadi and Nagaraja, 2022). Thus, improving the barrier health of the lower tract could be beneficial for helping reduce the prevalence of liver abscesses. In steers supplemented with BZ, ileal crypt depth tended to be shallower than CON in the current study. A decrease in intestinal crypt depth has been associated with increases in enzymatic activity that could result in improved nutrient absorption (Kelly et al., 1991) and decrease the amount of highly fermentable carbohydrates that could cause hindgut acidosis and lead to liver abscess formation.

## Conclusions

The inclusion of BZ into feedlot steers diets showed beneficial results during the receiving period and transition to a finishing diet. This indicates that cattle undergoing stress may benefit from BZ supplementation. While not statistically significant, the numerical differences observed over the cumulative finishing period warrant further investigation. Only minor positive effects of BZ supplementation were realized on carcass characteristics; however, intermediate supplementation of BZ for the reduction of liver abscesses and improving lower tract epithelial barrier integrity should be further investigated with improved statistical power based on the preliminary results of this trial.

## Data Availability

Data can be made available upon reasonable request to Z.K.S.

## Abbreviations

ADF: acid detergent fiber
ADG: average daily gain
AFBW: estimated body weight at 28% empty body fat
ASA: rumen wall absorptive surface area
BW: body weight
BZ: encapsulated butyric acid and zinc
CAFBW: carcass-adjusted final body weight
DM: dry matter
DMI: dry matter intake
DOF: days on feed
DRC: dry-rolled corn
EBF: empty body fat
EBW: empty body weight
EE: ether extract
EG: daily energy gain
EM: maintenance energy
G:F: feed conversion efficiency
GIT: gastrointestinal tract
HCW: hot carcass weight
MBW: median feeding period shrunk body weight
MDGS: modified distiller’s grains with solubles
MPA: mean rumen papillae area
MQ: maintenance coefficient
NDF: neutral detergent fiber
NE: net energy
NEg: net energy for gain
NEm: net energy for maintenance
NOP: rumen papillae per square centimeter
O:E: observed-to-expected
RFID: radio-frequency identification
SBW: shrunk body weight
SCFA: short-chain fatty acids
SERF: SDSU southeast research farm
W: mean equivalent shrunk body weight
YG: yield grade
